# Iceberg Indicators for Animal Welfare in Rural Sheep Farms Using the Five Domains Model Approach

**DOI:** 10.3390/ani10122273

**Published:** 2020-12-02

**Authors:** Rick Obrian Hernandez, Jorge Alberto Sánchez, Marlyn H. Romero

**Affiliations:** 1 Faculty of Agrarian and Animal Sciences, University of Caldas, Manizales 170004, Colombia; elrickhernandez@gmail.com; 2 Department of Animal Health, Faculty of Agrarian and Animal Sciences, University of Caldas, Manizales 170004, Colombia; Jorge.sanchez@ucaldas.edu.co

**Keywords:** sheep, five domains, iceberg indicators, animal welfare, smallholder

## Abstract

**Simple Summary:**

Animal welfare is difficult to quantify, especially among farmers, in extensive rural sheep farms where there is a low level of animal interaction and a lack of technology. In this study, we searched for iceberg indicators of animal welfare using the Five Domains Model approach, and studied the relationship among sheep flight distance, sheep handling training and job satisfaction in extensive rural sheep systems. A structured survey was used to obtain socio-demographical, job satisfaction and sheep handling training data. A full animal welfare evaluation was performed on all farms; furthermore, a health status examination was also made in which blood and stool samples were taken. Four iceberg indicators were found with the potential to predict overall animal welfare scores on farms, and one to study state of mind in extensive rural sheep systems, as well as interactions among job satisfaction, training in sheep handling and sheep mind state.

**Abstract:**

Animal welfare for sheep in extensive rural farms is difficult to quantify among rural farmers due to several factors, including the lack of technology and the low level of interaction they have with the animals. The purpose of this study was to search for animal-based iceberg indicators using the Five Domains Model approach and study the relationship between sheep reactive behavior (flight distance), sheep handling training and farmers job satisfaction. Thirteen extensive commercial dual-purpose sheep farms (*n* = 520 animals) were evaluated in Marulanda, Caldas (Colombia, South America). On-farm Animal Welfare Indicators (AWIN) were assessed using an adapted version of this protocol. Socio-demographic characteristics, sheep handling training and job satisfaction were evaluated using a structured interview. Blood and stool samples were taken to determine Fecal Egg Count and Packed Cell Volume. Bivariate regression models were used to find animal-based indicators that predicted Nutrition, Ambience, Health and Behavior welfare domains, and a Qualitative Behavior Analysis was used for mind state domain analysis. Body condition score (BCS) (*p* = 0.001), fleece cleanliness (*p* = 0.03), FAMACHA© Score (*p* = 0.05), and flight distance in meters (*p* = 0.19) were found to be indicators, and were useful for predicting overall welfare assessment (R2 = 0.85) on theses farms. Regarding mind welfare domain, Qualitative Behavioral Assessment found two principal components (PC) that explained 82% and 67% of the variance, and described emotional valence and energy levels of sheep, respectively. Sheep handling training (β = −8.75, *p* = 0.004) and job satisfaction (β = −7.5, *p* = 0.013) had a negative association with the average flock flight distance. Spearman’s rank correlations were significant (*p* < 0.001) between Fecal Egg Count, Packed Cell Volume, FAMACHA© Score (FS), Body Weight (BW) and, BCS. The strongest association was observed between Packed Cell Volume (PCV) and Fecal Egg Count (FEC) (*r* = −0.43), also FS was correlated with PCV (*r* = −0.28) and FEC (*r* = 0.21), and BCS was correlated with weight (*r* = 0.32). We suggest that these animal-based indicators could be useful as iceberg indicators for extensive sheep production systems and may set the ground for more research in small extensive sheep farms to develop strategies to find welfare problems and solutions.

## 1. Introduction

Traditional sheep farming is an important activity for rural producers, because it is a source of meat, wool, milk and skins, for their own consumption and family income. Governments, international organizations, nongovernmental organizations (NGOs) and producers have developed animal welfare standards for different purposes according to their respective interests: to encourage improving the animals’ quality of life, as regulatory health requirements to ensure food safety and as a strategy to promote and increase market share, among others [1]. Routine use of animal welfare indicators can help small-scale farmers assess the effectiveness of management practices, identify associated risk factors, and achieve better production outcomes [2].

There are several protocols for assessing the status of animal welfare in production systems, such as the Animal Welfare Indicators (AWIN) [3] and the Five Domains Model [4]. The AWIN protocol considers the four principles of animal welfare (good feeding, good housing, good health, and appropriate behavior) subdivided into 12 criteria developed by the European Welfare Quality program [3]. The Five Domains Model (nutrition, health, environment, health, behavior, and mental state) allows for a more detailed comprehension of the internal state of the animals and the external circumstances that affect animal welfare [4,5]. However, the implementation of animal welfare assessment protocols on farms requires expert and trained personnel. To be fully implemented, it can require between four and eight hours of work (depending on the species), which limits the number of farms that can be assessed in a given period, making this process costly and discouraging the commitment of producers to adopt them [6].

Animal welfare is about what animals experience, but there are also political, legal, trade, national and international policy, philosophical and social aspects to it [7]. In Colombia, the conflict between the government and a guerrilla movement (i.e., The Revolutionary Armed Forces of Colombia—People’s Army—FARC) ended in 2016 with a peace agreement. This process implied a lot of changes, not only political but also affecting land distribution and biodiversity in several regions [8]. For instance, people displaced from rural areas due to the conflict [9] had to start returning to their lands, and farmers who stayed, needed to start producing again. Nevertheless, this transitional period created a new set of uncertainties about the consequences of the new socio-political conditions and land use [10]. Changes in social dynamics and poverty level, one of the consequences of the conflict, as well as the lack of resources, threaten the sustainability, environmental conservation [11] and productivity of rural families. Sheep production systems are part of the agricultural sectors affected by post-conflict conditions in Colombia, mainly due to the traditional practices implemented in these systems and their geographic location, which limit the access to resources, and new knowledge and technology [12]. One of the initiatives for the social reincorporation of those affected by the armed conflict is the social reconstruction of their communities and the strengthening of their productive networks [13].

Likewise, regarding animal welfare, small sheep farm producers use traditional knowledge and require reliable, practical and easy to measure indicators under their productive conditions. It has been suggested that indirect indicators be identified and validated, that is, relatively simple measures that can reliably predict the results of other more complex or time-consuming indicators, which could improve evaluation protocols and significantly increase the probability of their adoption. These indicators have been called “iceberg indicators” that are not related in themselves to a specific problem, but can be used to indicate that a problem exists at the animal level and that a more detailed evaluation is required [6]. In accordance with the above, the objective of this article was to evaluate animal-based indicators with the AWIN protocols and the Five Domains Model, which allow the selection of possible iceberg indicators to facilitate evaluation and monitoring in traditional sheep farming systems, such as those affected by the armed conflict in Marulanda, Caldas, Colombia.

## 2. Materials and Methods

### 2.1. Location of Farms and Visits

**Figure 1:**
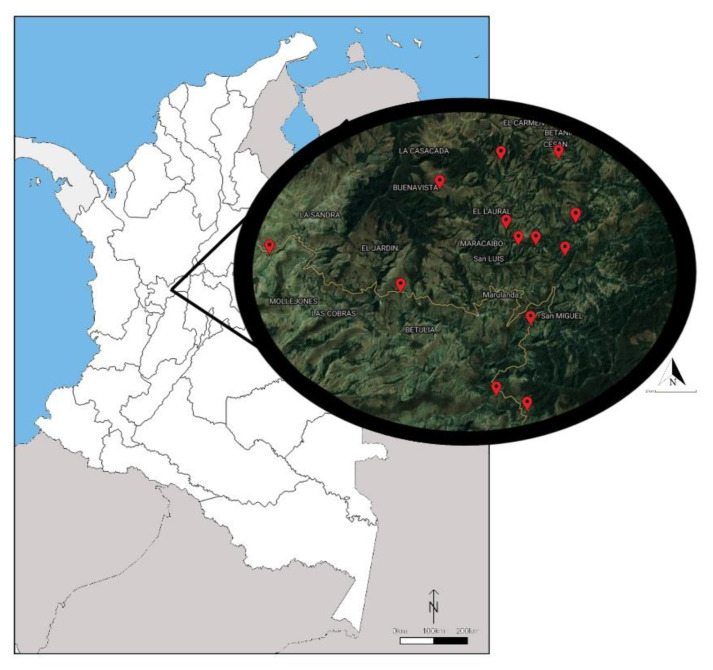
Geo-referencing of rural farms evaluated in Marulanda, Caldas, Colombia.

The study was carried out in the department of Caldas (western central Colombia) from August to November 2019 in all 13 commercial sheep farms that constitute the Sheep farmers’ Cooperative in Marulanda (5°17′03″ N 75°15′37″ O) (Figure 1). Marulanda is located in a cloud forest ecosystem with an average altitude of 2825 m and a mean yearly temperature of 13 °C. It is a region inhabited mainly by rural communities and people operating a local commerce economy.

### 2.2. Ethical Note

All procedures related to the use and care of the animals strictly followed Colombian laws and regulations, particularly Resolution 001634-2010, as stated by the Colombian Agricultural Institute [14]. Permission to conduct the study was approved by the Ethics Committee for Animal Experimentation (Act 1 25/08/2019)—Activities with minimal risk at the University of Caldas. Farmers were fully informed about the purpose of the study and the methods of stool and blood sheep sample collection, and they read/listened to and signed an informed consent form and authorization to allow us to use the data.

### 2.3. Characterization Survey

An ad hoc questionnaire was applied using a structured interview. The questionnaire was designed by the researchers in a focus group and divided into seven sections: geographical aspects, socio-demographic aspects, housing characteristics, productive system management, health management, feeding and production practices. Also answers about work environment and sheep handling training were gathered using another questionnaire applied at the same time using a 5-point Likert scale ranging from “strongly disagree” to “strongly agree” [15]. As not all farmers were able to read the questionnaire, interviewers read each question without any comment for all respondents and recorded their responses in categories from one (complete disagreement) to five (complete agreement). The information collected during the interview was handled anonymously during the analysis, interpretation and publication of the results. At the end of the study, all participants were informed of the full purpose of the study to prevent demand characteristics bias.

### 2.4. Animal Welfare Assessment

A total of 520 wool sheep were evaluated in the study. The number of animals evaluated was determined following a sample size calculation based on the total number of sheep at the farm as suggested by AWIN protocol guidelines (see AWIN, 2015 for more details) [16]. Sheep were managed under extensive commercial conditions and evaluated from 7:00 to 17:00, in other words, a full workday. The same observer applied a full AWIN sheep protocol on all the farms. A first evaluation was performed during grazing, where stereotypies, social withdrawal, excessive itching, panting, fleece cleanliness, fleece quality, tail length, lameness, flight distance, approximation, access to shelter, water availability and Qualitative Behavior Assessment (QBA) were evaluated. Then, a second evaluation was completed at a holding pen, where the observer could perform a closer inspection of the animals to be evaluated, this included: body condition score, head lesions, mucosa color, respiratory quality, body and legs lesions, fleece cleanliness, fleece quality, hoof condition, fecal soiling, mastitis, tail length, and lameness. The animal welfare score per farm was obtained following the AWIN guidelines, giving a percentage to each indicator according to the prevalence observed for it [16].

Body condition was evaluated by palpation of the lumbar area of the sheep to determine the musculature and fat layer present [17] using a scale of 1 to 5, where 1 represents a starving sheep/slender, while 5 represents a fat sheep [16]. The color of the mucosa was evaluated by the FAMACHA© method [18,19], which consists in comparing the observed color of the ocular conjunctiva with a reference scale printed on a card, whose values range from 1 to 5, where 1 represents a sheep with red mucosa (without anemia) and in optimal conditions, while 5 represents an anemic sheep [16]. The cleanliness of the wool was evaluated by direct observation of the presence of moisture and dirt in the areas covered with wool, except for the perianal region of the sheep [20,21]. A scale from 0 to 4 was used, where 0 corresponds to a dry and clean sheep, and 4 to a wet, dirty and muddy sheep in areas such as face, abdomen and flanks [16].

For the QBA, the total list of 21 descriptors listed in the AWIN protocol for sheep were exposed during a face-to-face focus group with four professionals to discuss their relevance and select the most suitable descriptors that have an expressive emotional connotation and provide information that is directly relevant to the welfare of grazing sheep. After this discussion, the group agreed on a final list of 15 descriptors to be used for performing QBA (with a fixed rating scale) on grazing sheep (Table 1).

The observer attended theoretical and practical training sessions, in which he scored all descriptors listed in the final list by observing videos of grazing sheep using the Visual Analogue Scale (VAS). A Qualitative Behavior Assessment (QBA) was the first thing done after arrival at the farms, and was performed during grazing and without any prior contact, clinical examination nor treatment on sheep. Descriptor scores were established according to the number of animals exhibiting the descriptor, the intensity of the descriptor and the interaction between the sheep [16]. The observer wore brown coveralls without any perfume or accessories to avoid altering the natural behavior of sheep. A total of four observation points, with a duration of five minutes per point, were used for each farm at an average distance of 50 m that did not disrupt sheep behavior but allowed a close observation of the whole flock.

### 2.5. Human Approach Test

The same observer, after the QBA assessment, performed a human reaction test at a grazing area by walking slowly towards the center of the flock in a straight line. When movement of the majority of sheep occurred, the observer stopped and recorded the flight distance in meters using stick markers placed prior to the test alongside the field fence with 2 m intervals or the fence post itself as a reference [22]. After that, the observer stood quietly for five minutes to check approximation from the sheep. If sheep actively walked towards the observer and interacted with him, e.g., sniffing, the observer recorded it as a one, otherwise as a zero. The flock of sheep used for this test had not undergone any painful or stressful procedures such as tail docking, castration or shearing, for at least 3 months prior to evaluation.

### 2.6. Blood and Fecal Sampling

A sample of blood from the jugular vein was taken from all assessed animals to determine Packed Cell Volume (PCV) values, which were measured using the micro hematocrit technique. Fecal samples were obtained directly from the rectum and were stored in refrigeration at 4 °C until analysis could be made for the determination of Fecal Egg Count (FEC) of Strongylidae spp. using McMaster Technique [23]. Body Weight (BW) [19] was also recorded at this instance. All samples were processed at the Animal Welfare and Parasitology Laboratory at The University of Caldas.

### 2.7. Statistical Analysis

All analyses were performed with STATA version 12.0 (StataCorp, College Station, TX, USA) using farms as the experimental unit. Descriptive statistics were calculated for socio-demographical data. Training and job satisfaction variables were transformed into categorical variables according to the number of training courses or sessions taken about sheep handling in the past two years (categories: 1, 2 or >2 courses) and the level of job satisfaction (1 = very low, 2 = acceptable, and 3 = very high job satisfaction) with the daily activities at farm. Spearman Rank correlations between FS, PCV, BW, BCS and FEC were made. Correlations were classified as very weak (*r* = ≤0.2), weak (*r* > 0.2 ≤ 0.4), moderate (*r* > 0.4 ≤ 0.7) and strong (*r* > 0.7) [24]. The distribution of normality of the AWIN animal welfare scores/farm was evaluated and a Student’s t-test was carried out to establish if significant differences existed between farms according to the flock size, taking into account that two farms were large (2000 and 600 animals) and 11 were small (range: 9–27 animals).

Animal welfare indicators were grouped according to the Five Domains Model, a score for each domain was obtained by weighted sum [25], and the overall welfare score corresponded to the average of four welfare principles (Nutrition, Ambience, Health, Behavior). Bivariate linear regression models per domain were made with the domain score as a dependent variable and prevalence of welfare indicators categories as independent variables. Coefficient of determination R2 and level of significance (*p* < 0.05) were used to select the best fitting models per domain. Selected indicators were used then as independent variables for another multivariate regression with the overall farm welfare score as the outcome and iceberg indicators remained in the model based on their level of significance (*p* < 0.05) and the adjusted coefficient of determination R2.

The mind welfare domain was evaluated through a Principal Component Analysis (PCA) performed on QBA descriptors scores for each farm. Principal components with Eigen values >1 were selected (created as new variables) and used as independent variables for a bivariate linear regression model with the average sheep flight distance per farm as the outcome as well as another model to predict how farmer’s job satisfaction and sheep handling training affect sheep flight distance. Variables including panting, lamb mortality, access to shelter, head and body lesions, lameness, respiratory quality, water availability, excessive itching, stereotypy, and udder lesions showed little variation (>80% of the observations corresponded to zero values); therefore, they were excluded from the analysis.

## 3. Results

### 3.1. Characterization Survey

All farmers were male, with an average age of 46 years, and an average of 27 years of work experience with sheep farming. Regarding marital status, two farmers were single, four had a common-law partner, and seven were married. More than half of the farmers had elementary school education while four had high school education, and two had a college degree. All producers were affected by the armed conflict, eight of whom were direct victims due to forced displacement from their lands. Farmers had access to free sheep handling and production management courses through government institutions or programs. In the last two years, two farmers attended three or more of these training courses, seven attended two training sessions, while four attended one or less formal training courses in sheep management or handling in the same period.

Regarding work environment, all farmers reported good relationships with coworkers, since most of them were relatives that usually did not live at the same house or family members. Nine farmers reported being worried about safety conditions and risk management in their job, especially for injuries at sheep handling maneuvers.

The median of flock size on the farms was 17 sheep (range: 9 to 2000 sheep). The main breed was Romney marsh, followed by Colombian Moro and Suffolk. The average farm size was 200 ha (range: 30 to 520 ha). All farms used extensive grazing systems and were classified as dual-purpose farms. Table 2 describes characteristics by farm. In all farms, the owner and their family performed all animal handling and care activities, as there was no contracted labor. All farms lacked production records for birth, mortality, grazing areas management, or health treatments.

Only two farmers using an emasculator on lambs performed castration, eight farmers performed tail docking with a knife and hot iron, and only four farmers performed navel dip disinfection of the newborn lambs with iodine solutions. All farmers disposed of dead sheep by burying them in grazing land. Animal identification was found on 11 farms, with seven using ear notch and four using ear tags. For castration and tail cutting procedures, none of the farmers used anesthesia or pain relief methods, and shearing was undertaken with scissors on floors or rudimentary tables.

### 3.2. Animal Welfare Assessment

Overall, farms had a mean welfare score of 64.1 ± Standard deviation (SD): ± 7.3%; range: 52.9, to 78.5%, only two farms had a welfare score below the 54% threshold of acceptable welfare levels [16]. However, a large percentage of sheep presented a body condition ≤2 and a FAMACHA© index ≥4, which shows that the domains of nutrition and health were greatly affected on the farms. Additionally, no statistically significant differences were found when comparing animal welfare scores between the two groups of large farms (600 and 2000 animals) and small farms (9 to 27 animals).

Regarding animal-based indicators (Table 3), a total of 520 sheep were evaluated among all farms, and 80.2% (*n* = 417) had a BCS ≤ 2 and 22.9% (*n* = 119) showed substantial dirt in their fleece (fleece cleanliness score ≥ 2). Sixty-four per cent of the evaluated sheep (*n* = 335) presented fecal soiling (fecal soiling score ≥ 1) and 34.6% (*n* =180) had ocular discharge. Regarding FAMACHA© score, 16.1% of the animals (*n* = 84) presented a score of ≥4, while 52.8% (*n* = 274) showed a score of ≤ 2. In relation to flight distance, the average was 8.7 m (±4; range 0 to 15 m). Only one farm reported a 0 m of flight distance to a non-familiar human approaching and it was the only farm that presented approximation of sheep. Bivariate linear regression models identified one animal-based welfare indicator for each of the first four domains (Nutrition, Environment, Health, and Behavior) that had a direct relationship with the overall on-farm welfare score (Table 4). These indicators were BCS (R2 = 0.29; *p* = 0.03) for the Nutrition domain, fleece cleanliness score (R2 = 0.61; *p* = 0.01) for the Environment domain, FAMACHA© score (R2 = 0.34, *p* = 0.02) for the Health domain, and finally, flight distance (R2 = 0.76, *p* < 0.0001) for the Behavior domain. In the multivariate regression model only BCS (*p* = 0.001), fleece cleanliness (*p* = 0.03) and FAMACHA© score (*p* = 0.05) were significant and explained 85% of the overall welfare outcome per farm. Flight distance, although not significant, was kept in the model as a confounding variable.

### 3.3. Qualitative Behavioral Assessment—Mind Domain

Concerning the state of mind domain (fifth domain), Principal Components Analysis for QBA of all farms identified two main factors with eigenvalues greater than 1 (9.49, 2.05). They accounted for 82% of the variance. The first principal component (PC1) accounted for 67% of the variance and this dimension was characterized as “content” versus “fearful” and appears to distinguish between sheep’s emotional valence. The second principal component (PC2) accounted for 14% of variance, and was characterized as “relaxed” versus “vigorous”, and characterized the energy level in sheep. Table 5 shows the factor loading of principal components for QBA descriptors evaluated.

PC1 (emotional valence) and PC2 (energy level) were associated significantly with average flight distance per farm, specifically, a sheep flock labeled as “Fearful” or “Vigorous” presented greater flight distance. Additionally, the number of handling trainings courses for sheep production taken in the last two years and the job satisfaction status of farmers also affected flight distance, showing that when farmer training increases and job satisfaction levels are high, sheep flight distance diminishes (Table 6).

### 3.4. Associations between Fecal Egg Count, Packed Cell Volume and Animal-Based Welfare Indicators

There was a significant negative correlation between FEC and PCV, as well as with BCS and weight. Additionally, FEC and FAMACHA© score were positively correlated. On the other hand, PCV was positively correlated with BCS and weight, and negatively correlated with the FAMACHA© score. There was a significant positive correlation between BCS and weight (Table 7).

## 4. Discussion

Iceberg indicators for animal welfare are key factors for the identification of welfare problems on farms [6,26]. It is important to understand the relation between iceberg and animal-based welfare indicators and design monitoring methods to assess animal welfare quickly and effectively on small sheep farms, which usually do not have the monetary resources to hire trained specialists for this. Several studies have searched for these indicators in cattle [27] and pigs [28], but no previous studies have been published about this type of indicators for sheep in extensive systems. To our knowledge, this is the first study to examine iceberg indicators for extensive sheep production systems managed by rural farmers in a socio-political post-conflict zone. While the results of this study might be more representative for small rural farms with extensive sheep systems, the principal type of sheep production system in Latin America, the findings of this study set up a precedent and provide valuable insight into sheep welfare evaluation for small farmers worldwide in similar conditions.

### 4.1. Farm and Producer Features

Worldwide, extensive management systems are the most common type of sheep production [29], and this is not an exception for Colombian systems, where sheep farms are located in steep mountain areas in cloud forest ecosystems, where other agricultural and livestock activities are performed as well [30]. The lack of technology, basic traditional management without appropriate records, and thus, inappropriate decision-making, as well as low incomes, are the common denominators of Colombian sheep production systems [14,26]. These same conditions were found in the sheep farms evaluated, where it was observed that poor decision-making was carried regarding the selection and reproduction of sheep, whereby the farmer focuses on selecting animals that are adapted to the local environment and capable of surviving and growing with little intervention and care by the farmer and the veterinarian. Under these conditions, the lack of care for newborn lambs has caused a reduction in flock size, especially due to predation by wild dogs (*Canis familiaris*) and cougars (*Puma concolor*) [28].

Farm conditions in this study were similar to those seen in Africa and Asia [31,32], where the main characteristics are small flocks, extensive grazing systems with a lot of land available, the presence of predators, and lack of vigilance and monitoring by the farmers. Predation by carnivores was reported anecdotally by all farmers in the present study, but the lack of records about the attacks made it impossible to include it in the analysis. All farmers were men, although small ruminant production systems in developing countries like India and Latin American countries have been actively incentivized for women due to their easy management [33]. Producer education level was low, but no significant correlation was found in this study between formal education and sheep welfare, as was the case with training.

### 4.2. Animal Welfare Assessment

Overall, most sheep farms in this study had a “good” welfare score for the environment domain, with access to shade and shelter, fleece cleanliness, and panting indicators, along with the behavior domain with stereotypes, excessive itching, social withdrawal, and flight. While the nutrition domain, with the prevalent BCS ≤ 2, no water availability and high lamb mortality reported due to predation and the health domain with fecal soiling, FAMACHA© scores ≥ 4, and ocular discharge had lower average scores. In this study, no significant differences were found for animal welfare classification between the two large farms and the eleven small farms. This observation may be related to the socio-political conditions of the armed conflict, which kept these areas geographically isolated, without state presence, and subjected them to forced displacement. For more than fifty years, affected rural producers did not have access to technology and knowledge sharing related to management practices and veterinary assistance [10,34], which affected producers in the area, as evidenced in the study by the absence of basic production records (records for birth, mortality, etc.), traditional management practices, and a lack of professional assistance.

A study performed in Mexico [35] suggests that small flock sheep farmers due to low density, easiness of handling, and family managed systems have an advantage in achieving good animal welfare over larger production systems. However, in these small and extensive systems, the domains of Behavior and Environment tend to be favored due to the lack of space constrains and a free roaming environment. Nonetheless, the domains of Nutrition and Health can be negatively impacted due to the lack of monitoring, so overall welfare may be affected by this unbalanced relation [36]. It has also been reported that cultural differences among producers have an impact over production parameters in small farmers due to management and the availability of resources [37].

### 4.3. Iceberg Indicators

This study found that BCS, FAMACHA© score, fleece cleanliness, and flight distance had a significant impact on the overall on-farm welfare score, and QBA represented the mind state of sheep (mental domain). These indicators have been reported to be used all around the world for extensive sheep systems and have been proven as reliable, feasible, and valid [38]. Low BCS measured through palpation at the lumbar section was the best indicator identified in this study to evaluate the Nutrition welfare domain, being BCS ≤ 2 the most prevalent value found in all farms. This could be explained due to the fact that the nutritional value of pastures on extensive hill systems are normally not adequate and the nourishment of animals is sub-optimal [39,40].

This study found fleece cleanliness to be a significant indicator for the environment welfare domain, as it helps to determine when small rural extensive farms with sheep in all year outdoor conditions have no protection against extreme weather, which can affect thermoregulation and energy consumption [37]. It has also been reported that its measurement presents high inter-observer agreement in extensive farming systems [20], which makes it useful to evaluate changes throughout all production stages on farm.

Regarding the Health welfare domain, we identified that the FAMACHA© score (FS) ≥ 4 (a.k.a. anemic sheep) had an impact on the overall welfare score. Generally, FS is related to gastrointestinal parasite infestation. Since it has been reported that gastrointestinal parasite prevalence is common in sheep productions worldwide [41,42] it is important to detect them prematurely in order to avoid economic and productive losses; however, in this study, the correlation between FEC and FS was low (*r* = 0.21). This is because the relation is highly dependent on the presence of *Haemonchus contortus*, and FS is not sensitive to other parasitic burdens [18]. Therefore, ideally, FA has to be measured along with BCS or weight to improve its effectiveness. In addition, FAMACHA© is a subjective indicator that depends strictly on the appreciation of the mucous color by the observer, so in order to use this properly and as a reliable indicator, proper training is required [43,44].

With regard to the evaluation of the animals’ mental states, the QBA is considered a qualitative measurement scale used and validated in ovine [45], bovine [25], porcine [46], and equine species [47]; however, to avoid measurement biases, adequate instruction and training in the use of the visual analog scale and the interpretation of the descriptors is required [48]. In the present study, this factor was controlled by the training of a postgraduate veterinary doctor with training in animal welfare. There are quantitative methods for behavior assessment, such as the open field test or the new object test, that have been used in experimental conditions and have proven to be useful for the assessment of mental states and temperament in animals [49]; however, they need to be validated for sheep species under field conditions.

For evaluated farms, on average long flight distances (average 8.7 ± 4 m) were found, this is a common situation in sheep production systems where painful procedures, such as tail docking and castration, are performed without anesthetics or pain relief methods [50]. Only one of the farms in this study had no flight distance but positive approximation to the observer; this farm did not do any castration or tail docking procedures, and the farmer kept close contact with his sheep. In addition, handling procedures such as deworming, transport and identification use a variety of physical and sound signals, which increase the sheep’s reactivity and affect human–animal interactions [51]. Shearing has also been reported to be a major cause for increased reactivity and stress in wool sheep, due not only to the handling procedure itself but also for the energy consumption necessary for thermoregulation after shearing [52], especially in high lands with low temperatures such as the ones in this study.

There were relationships between reactivity, energy level, sheep handling training and job satisfaction and flight distance in this study. Regarding the amount of training received by producers (in the last 2 years), we found that as the amount of handling training increased, average flight distance decreased. This result is similar to findings in other studies that have demonstrated that livestock handling training is directly correlated with behavioral indicators and easiness of handling in abattoirs and farms [53]. This suggests that proper training of farmers may lead to an improvement in reducing the stress produced in situations such as shearing. Knowledge about animal welfare itself does not avoid bad handling practices or bad human-animal interaction experiences, since even when people have knowledge about animal welfare, their attitude towards animals can be negatively influenced by their own internal perceptions of the world [47]. Whatever the mechanism by which job satisfaction in farmers significantly reduced the flight distance in their sheep, one possible explanation could be that there is a known relationship among farmers’ or handlers’ behavior toward animals that is influenced by the attitude and personality of the farmers [54]. Farmers in post-conflict areas suffered from violence that affected their lives in many respects. Besides displacement, the physiological trauma and emotional distress [55] negatively affected farmers’ mental health in a scenario where almost ten percent of adults in post conflict areas suffer from a mental health issue such as depression, post-traumatic stress disorder, and anxiety [56]. Therefore, since farmers and stock people have the greatest influence on animal welfare [51], training that involves all these dimensions is required to positively affect animal welfare.

Our findings present some key aspects for small flock rural farmers that could have an important effect on animal welfare problems on farms, and some iceberg indicators to serve farmers as a tool to study and compare animal welfare over time. Since high hill, extensive grazing lands tend to lack good pastures and have low average BCS, as long as farmers have proper training, this could be a powerful tool for quickly assessing the nutritional state of animals as an aid to determine when to implement solutions to ensure that sheep obtain all nutritional requirements and improve both production and welfare. Meanwhile, extensive rural systems tend to lack shelter, whether natural or man-made, for protecting sheep against extreme weather conditions. Furthermore, average fleece cleanliness allows farmers to identify protection from environmental conditions during any handling procedure. We suggest that farmers could use silvopastoral systems to provide natural shelter for sheep kept outdoors that will help to diminish this problem while improving animals’ nutrition [57,58].

Low contact with sheep in these extensive systems could lead to health problems to go unnoticed, until they affect production and welfare parameters, for instance gastrointestinal infestation (i.e., *Haemonchus contortus*) as one of the prevalent conditions for sheep production systems. FAMACHA© allows farmers to detect anemic sheep (Score ≥ 4) so they can be selected and dewormed during handling procedures; however, FAMACHA© requires proper training to use on farms, since it relies on subjective color appreciation of mucosa.

Flight distance, as well as QBA, reflected the mind state of sheep, which is affected mainly by the farmer’s attitude, training and knowledge about animal welfare, and the production procedures such as tail docking, identification, or castration, all of which are affected by the farmer’s environment and traditions. The interactions among farmers and livestock should not be studied isolated from their cultural and social background. In fact, good understanding of culture and background will help to create specialized training for farmers that target their key characteristics to reduce flight distance and ease handling to improve productivity [59].

According to this, the next step in this program and further studies to impact small sheep farmers in extensive production systems are: first, to promote and evaluate different methods for production record management that allows for constant monitoring of extensive production systems. Second, to perform and evaluate the impact of training sessions for farmers to use iceberg animal welfare indicators consistently and accurately to allow them to identify early welfare problems in their farms; and last but not least, design and evaluate training strategies for sheep handlers taking into consideration their key socio-demographic characteristics to find the best approach to improve Human-animal interactions.

Finally, we found BCS, fleece Cleanliness, flight distance and FAMACHA© to be iceberg indicators that explain 85% of variance of the overall welfare score, and QBA to be an important tool for inspecting and assessing the mind state of sheep on-farm. The 22 indicators for the AWIN sheep protocol were reduced to five; this reduction allows for a practical and quick animal welfare assessment by small farmers on extensive sheep production systems. In addition, we found interactions with respect to sheep handling training and job satisfaction to be key aspects for interventions to improve human animal interactions and sheep handling for extensive rural sheep farmers.

## 5. Conclusions

Assessment of body condition, wool cleanliness, FAMACHA©, flight distance and qualitative behavior analysis are suggested as iceberg indicators. They can be useful to quickly and generally assess the level of welfare in extensive smallholder sheep systems. These indicators, when evaluated routinely, can help to identify problems of animal welfare, assess the effectiveness of management practices in place, guide intervention programs or improve the level of animal welfare on sheep farms. Furthermore, over time, they can be used to monitor and evaluate the sanitary measures in place, and identify risk factors for specific health problems, such as the presence of endo-parasites, applying FAMACHA© index. Considering the difficulty of implementing more complex protocols to evaluate animal welfare, these indicators can identify problems associated with the nutritional, environmental, health, behavioral and mental domains of sheep in a timely manner.

## Figures and Tables

**Table 1:** Selected descriptors and definitions for the QBA assessment in grazing sheep, Marulanda, Caldas (Colombia).

Descriptor	Definition
Alert	Observant and Vigilant
Active	Sheep is physically active, engaged in task.
Relaxed	At ease, free from anxiety, agitation or tension, the sheep appears to be unthreatened
Content	Satisficed and at peace. The sheep’s needs are met or the sheep is working towards their fulfillment
Sociable	Seeking and interacting with other sheep. The sheep seems to be enjoying/taking comfort from its contact. The sheep chooses to be part of the flock and not fully isolating itself
Joyful	Sheep is happy, comfortable, at ease, satisfied about its environment, playful. It may play with other group members.
Fearful	Attention is focused on one specific object/being, which is either a real or a perceived threat. Animal may also be fleeing
Wary	Shy, cautious, apprehensive and possibly distrustful
Tense	Uneasy and/or on-edge. Posture may show physical tension.
Aggressive	Hostile and tense. Attacking/ready to attack, usually unprovoked or competing for a resource
Agitated	Excessive cognitive and/or motor activity due to tension or anxiety. The animal is uneasy, and if moving their actions are twitchy.
Vigorous	The animal is carrying out tasks in an energetic or forceful way. If stationary or moving slowly, the animal expresses an inner strength and energy. May imply good physical health.
Physically Uncomfortable	Giving impression of pain or other physical discomfort through posture/movement.
Frustrated	Dissatisfied. Unable to fulfil satisfaction and achieve goals.
Apathetic	Unresponsive and dull

**Table 2:** Altitude in meters above sea level (masl), flock size (ewes > 1 year old), extension in hectares, AWIN total welfare score and other livestock species present for 13 evaluated farms.

Altitude (masl)	Flock Size	Extension (ha)	Welfare Score, %	Other Species
2749	11	30	54, 7	Cattle
2663	21	32	67, 6	Cattle
2690	10	80	65, 3	Cattle, chickens
2485	23	84	57, 9	Cattle
2500	10	100	74, 5	Cattle
2434	9	130	65, 9	None
2987	17	111	61	Cattle, chickens
3031	27	144	52, 9	None
2963	21	240	67, 4	Cattle, Pigs
2902	14	350	78, 5	Cattle, horses
2650	15	350	63, 5	Cattle
3046	600	432	60, 9	Cattle, horses
2825	2000	520	58, 2	Cattle, horses

**Table 3:** Percentage, standard deviation (SD), minimum and maximum number scored for each category of animal-based welfare indicators.

Indicator	Category	Percentage (*n* = 520)	SD	Minimum	Maximum
Body condition score	1	43.9 (228)	±19.1	0	74.0
	2	36.3 (189)	±17.8	10	75
	3	16.3 (85)	±14.0	0	42.8
	4	3.3 (17)	±8.1	0	28.5
	5	0.2 (1)	±0.7	0	2.6
Fleece Cleanliness	0	48.5 (252)	±36.8	0	100
	1	28.6 (149)	±23.9	0	70
	2	11.8 (61)	±21.3	0	66.6
	3	5.3 (28)	±8.5	0	22.2
	4	5.7 (30)	±20.5	0	74.0
Fecal soiling	0	35.7(185)	±30.5	0	100
	1	30.4 (158)	±22.8	0	90
	2	16.7 (87)	±14.0	0	40.7
	3	12.6 (65)	±13.60	0	40
	4	4.5 (25)	±9.4	0	30.4
Fleece Quality	0	95.5 (496)	±7.6	78.1	100
	1	3.4 (19)	±6.2	0	21.5
	2	1.1(5)	±3.9	0	14.2
Ocular Discharge	0	65.5 (340)	±18.4	33.3	92.8
	1	32.7 (169)	±20.7	0	66.6
	2	1.9 (11)	±6.9	0	25
FAMACHA^©^ Score	1	19.3 (100)	±15.7	0	47.8
	2	33.5 (174)	±15.6	4.7	66.6
	3	31.1 (162)	±16.5	14.8	75
	4	13.7 (71)	±16.2	0	57.1

**Table 4:** Bivariate linear regression models showing animal-based indicators significant for Nutrition, Ambience, Health and Behavior domains, and a multivariable model evaluating factors associated with the overall on-farm welfare score.

Variables	Nutrition			Ambience			Health			Behavior			Welfare Score		
	R^2^ = 0.29			R^2^ = 0.61			R^2^ = 0.34			R^2^ = 0.76			R^2^ = 0.85		
	*ß*	*SE*	*p*	*ß*	*SE*	*p*	*ß*	*SE*	*p*	*ß*	*SE*	*p*	*ß*	*SE*	*p*
Body Condition Score 1 ^1^	−0.31	0.12	0.03	-	-	-	-	-	-	-	-	-	−0.27	−0.04	0.001
Fleece cleanliness score ≥ 3 ^2^	-	-	-	−0.62	0.13	0.001	-	-	-	-	-	-	−0.30	0.11	0.03
FAMACHA© Score ≥ 4 ^3^	-	-	-	-	-	-	−0.26	0.10	0.02	-	-	-	−0.14	0.06	0.05
Flight Distance (m) ^4^	-	-	-	-	-	-	-	-	-	−0.82	0.12	≤0.001	0.05	0.04	0.19

^1^ Prevalence (%) of sheep ≥1 year old with body condition score = 1; ^2^ Prevalence (%) of sheep ≥1 year old with fleece cleanliness ≥3; ^3^ Prevalence (%) of sheep ≥1 year old with FAMACHA© score ≥4; ^4^ Confounder variable for body condition score and FAMACHA© score.

**Table 5:** Loadings of principal components (PC) for Qualitative Behavioral Assessment (QBA) descriptors used in to evaluate welfare of grazing sheep.

QBA Descriptors	PC1	PC2
Alert	−0.62	0.42
Active	−0.41	0.80
Relaxed	−0.90	−0.31
Content	−0.98	−0.07
Sociable	−0.90	−0.20
Vigorous	−0.43	0.86
Joyful	−0.94	−0.20
Fearful	0.97	−0.07
Wary	0.76	−0.18
Tense	0.87	0.13
Agitated	0.86	−0.2
Physically uncomfortable	0.82	0.39
Frustrated	0.94	0.19
Apathetic	0.70	0.07

**Table 6:** Multivariable linear regression models evaluating the association of the average flock flight distance (outcome) with the behavioral principal components (model 1), that characterized valence (PC1) and energy (PC2) of the flock of sheep and the level of job satisfaction perceived by producers and the number of training courses taken by the producer in the last 2 years (model 2).

Model and Variables	Sheep Flight Distance, m		
	ß	SE	*p*-Value
*Model 1*, R^2^ = 0.84			
PC1 (Fearful–Content)	23.03	2.98	<0.001
PC2 (Vigorous–Relaxed)	7.30	2.99	0.03
*Model 2*, R^2^ = 0.78			
Training Sheep Handling			
1 *	−4.5	1.3	0.011
2	−5.25	1.6	0.014
3	−8.75	2.07	0.004
Job satisfaction			
1	−1.36	4.55	NS
2	−2	6.17	NS
3 *	−7.5	2.26	0.013

* Reference Category. NS: non-significant.

**Table 7:** Spearman Rank’s Correlations between animal-based welfare indicators and Fecal Egg Count (FEC) and Packed Cell Volume (PCV).

Variables	FS	PCV	FEC	BW	BCS
FS	1.0				
PCV	−0.28 **	1.0			
FEC	0.21 **	−0.43 **	1.0		
BW	−0.11 **	0.29 **	−0.29 **	1.0	
BCS	−0.23 **	0.28 **	−0.17 **	0.32 **	1.0

** *p* < 0.001. FS: FAMACHA© score. BW: Body Weight. BCS: Body condition score.
